# Electrophysiological correlates of respiratory failure in acute organophosphate poisoning: Evidence for differential roles of muscarinic and nicotinic stimulation

**DOI:** 10.3109/15563650.2012.670875

**Published:** 2012-03-29

**Authors:** Pradeepa Jayawardane, Nimal Senanayake, Nick A Buckley, Andrew H Dawson

**Affiliations:** 1 Department of Pharmacology, Faculty of Medical Sciences, University of Sri Jayewardenepura, Sri Lanka; 2 South Asian Clinical Toxicology Research Collaboration, University of Peradeniya, Sri Lanka; 3 Professorial Medicine Unit, POW Clinical School, University of New South Wales, Sydney, Australia; 4 Royal Prince Alfred Clinical School, University of Sydney, NSW, Australia

**Keywords:** Neurophysiology, Ventilation, Organophosphorous compounds

## Abstract

*Background.* Respiratory failure in acute organophosphate (OP) poisoning can occur early and also relatively late in the clinical course, and the pathophysiology of respiratory failure at these different phases may have important clinical implications. *Objective.* To compare the electrophysiological findings in patients with early and late respiratory failure following acute OP poisoning. *Methods.* A prospective observational case series of consenting symptomatic patients with acute OP poisoning were assessed with daily physical examinations and repetitive nerve stimulation (RNS) studies. RNS was done on right and left median and ulnar nerves at 1, 3, 10, 15, 20, and 30 Hz. Outcomes such as need for ventilation and development of intermediate syndrome (IMS) were noted. Early respiratory failure was defined as occurring within 24 hours of ingestion. *Results.* Seventy-eight patients were recruited for the clinical and electrophysiological study and of those 59 (75.6%) patients had ingested chlorpyrifos. Seven patients developed respiratory failure within 24 hours of ingestion with overt muscarinic signs. They had no electrophysiological abnormalities at median and ulnar nerves before intubation. Three of them later developed “forme fruste” IMS. Five other patients developed late respiratory failure after 24 hours of ingestion, and all of them showed progressive RNS changes indicating severe IMS prior to intubation. *Conclusion.* The normal RNS in all patients developing early respiratory failure suggests that it is due to a central nervous system (CNS) and muscarinic effect. This emphasizes the need for early rapid atropinisation as a priority, combating the nicotinic effects being less urgent. This is in contrast with the late respiratory failure, which has been shown to be associated with neuromuscular dysfunction. Further studies are needed to quantify CNS and muscarinic dysfunction to assist in the development of better treatments for the severe and early OP poisoning.

## Background

Acute organophosphate (OP) poisoning is a major global health problem^1^,^2^ with hundreds of thousands of deaths every year.^3^,^4^ Respiratory failure is a significant cause of the high morbidity and mortality from acute OP poisoning, especially in resource poor settings in developing countries.

Respiratory failure occurs with two relatively distinctive clinical patterns. An early form occurs at or soon after admission in patients with impaired level of consciousness and with marked muscarinic features.^5^ Late respiratory failure typically occurs in conscious patients whose muscarinic symptoms are controlled and is attributed to weakness of the respiratory muscles due to intermediate syndrome (IMS).^6^,^7^ There are many potential mechanisms of respiratory failure following acute OP poisoning. Animal studies support the idea that early OP-induced respiratory failure results from effects of OPs on muscarinic brainstem circuits, thereby interfering with respiratory rhythmogenesis, and on lung tissues thereby affecting secretary, airway, and vascular function.^8^ The main objective of this study was to compare the electrophysiological findings associated with early and late respiratory failure in humans.

## Methods

A prospective observational study of symptomatic OP poisoned patients was carried out in the Central Province of Sri Lanka with the approval of the Ethics Committees of the University of Peradeniya, Sri Lanka and the Australian National University, Canberra, Australia. Patients were recruited from General Hospital, Nuwara Eliya from May 2005 to April 2006 and from Teaching Hospital, Peradeniya from May 2006 to December 2006. All patients were assessed on admission and repeatedly thereafter for features of acute cholinergic syndrome by the first author and MBBS qualified clinical research assistants. The inclusion criteria were admission within 24 hours of ingestion of OP and signs of systemic intoxication. Patients under an age of 15 years and pregnant patients were excluded. Informed written consent was obtained from all the study patients. If the patients were unconscious, consent was obtained from the accompanying relatives, but the consent was confirmed with the patients themselves when they regained consciousness. OP poisoning was confirmed by the history from the patient and/or relatives, containers brought to hospital, information on patient-transfer forms, characteristic smell in the breath, and clinical features typical of OP poisoning. OP concentration in plasma was quantified in 67 of 78 patients using reversed phase high performance liquid chromatography and ultraviolet detection. Red blood cell acetylcholinest-erase (RBC AChE) level was assessed in 59 of 78 patients using the modified Ellman method.^9^ Accordingly, additional biochemical evidence of OP poisoning (serum OP level or RBC AChE level) was available in 69 of the 78 patients. As per institutional practises, the patients were treated with 10–15 mg bolus dose of atropine followed by 10–15 mg IV atropine infusion in 0.9% normal saline over about 12 hours. The infusion rate was adjusted according to the patient's clinical situation. If the patient became excessively atropin-ized, the infusion was discontinued. If the patient developed further cholinergic features, further boluses (e.g. 10–15 mg IV) were given. Pralidoxime was administered 1 g q 6 h by slow IV injection for 48 h.

### Clinical and electrophysiological assessment

All the patients were assessed at least twice daily with a focused neurological examination to detect signs of IMS and at least six times a day for cholinergic (nicotinic and muscarinic) signs. Bedside electrophysiological testing was carried out by the first author using a portable Medelec Synergy electromyography machine (software version 11). The first assessment was done within 24 hours of poisoning in 69 of 78 patients. The studies were repeated daily until there were no detectable electrophysiological or clinical abnormalities. Repetitive nerve stimulation (RNS) was performed on right and left median and ulnar nerves. Single supramaximal stimulation of the same nerves was done to detect repetitive responses. Nerves were stimulated superficially by a stimulator fixed to the respective nerves at the wrist. Recordings were done with TECA NCS disposable bar electrodes using the belly tendon configuration. The “stimulation” hand was immobilized manually to prevent movement artifacts. A 50-Hz notch filter was used. RNS studies were done with train of 10 supramaximal stimuli of 0.1 millisecond at 1, 3, 10, 15, 20, and 30 Hz frequencies. There was at least a 15 seconds interval between two trains of stimuli. In normal RNS studies, the amplitudes of compound muscle action potentials over the train do not differ. In some, at high-frequency stimulations, incremental patterns are observed where the amplitude of the compound muscle action potentials seems to increase progressively over the train. This increment is a normal variant and is described as a pseudoincrement. The main abnormalities observed on RNS in OP are decrement-increment and progressive decrement patterns.^10^ The electrophysiological abnormalities of the cohort have been described previously.^11^

Data are presented with conventional nonparametric descriptive summary statistics.

## Results

All 78 patients had muscarinic and nicotinic symptoms at enrolment. Sixty-five of the 78 patients were males. Fifty-nine patients had ingested chlorpyrifos. The other patients ingested dimethoate (2), phenthoate (1), and diazinon (1) and unknown OP (15).

Twelve (15%) patients developed respiratory failure requiring mechanical ventilation. Early respiratory failure (within 24 hours) was associated with a lower Glasgow Coma Scale (GCS) and a shorter duration of ventilation compared to those developing respiratory failure later (Table 1).

**Table 1:** Characteristics of patients developed early respiratory failure and late respiratory failure.

	Early respiratory failure (median and range) *n* = 7	Late respiratory failure (median and range) *n* = 5
Age (years)	22 (18–56)	25 (18–29)
Male:female	5:2	2:3
Type of OP	5 Chlorpyrifos	1 Chlorpyrifos
	1 Phenthoate	1 Dimethoate
	1 Unknown	3 Unknown
Time since ingestion to intubation (hours)	6 (2–17)	86 (24–95)
Admission		
GCS	11 (3–15)	15 (9–15)
SBP (mmHg)	120 (90–140)	117 (110–140)
Pulse (BPM)	60 (60–92)	80 (60–120)
On intubation		
GCS	10 (4–13)	15 (12–15)
SBP (mmHg)	120 (90–130)	100 (100–130)
Pulse (BPM)	110 (72–120)	76 (60–120)
Duration of ventilation (hours)	99 (2–203)	208 (93–718)

OP, organophosphate; GCS, Glasgow Coma Scale; SBP, systolic blood pressure; BPM, beats per minute.

Seven of the 12 developed respiratory failure within 24 hours when they were acutely ill in severe cholinergic crisis (early respiratory failure). The important clinical features at the time of intubation of these seven patients are described in Table 1. Five patients developed respiratory failure more than 24 hours after OP ingestion (late respiratory failure). None of the patients had a GCS at the time of intubation of less than 12, indicating central nervous system (CNS) depression was not a major factor in the respiratory depression. All five patients had severe muscle weakness (Medical Research Council grade 3 or less) involving the proximal limb muscles and neck flexors with weakness of the muscles supplied by the motor cranial nerves confirming IMS. One of these patients died when she was still in respiratory failure 9 days following admission. Features of those five patients at the time of intubation are described in Table 1.

Frequent electrophysiological studies were performed from admission in all 12 patients to monitor neuromuscular transmission. The patients who developed early respiratory failure had no electrophysiological evidence of neuromuscular transmission failure around the time of intubation. However, three of the seven developed a mild degree of weakness and decrement-increment patterns at high frequency stimulations after 24 hours as seen in the “forme fruste” IMS.^11^

All patients who developed respiratory failure after the first 24 hours demonstrated electrophysiological abnormalities of progressive neuromuscular transmission failure,^10^,^11^ and at the time of intubation they had severe decrement or progressive decrement patterns at high frequency stimulations (20–30 Hz) suggesting severe disruption of neuromuscular transmission.

## Discussion

The mechanisms of respiratory failure following acute OP poisoning are complex. Many patients develop respiratory failure in the early stage of severe acute poisoning. In 1987, Senanayake and Karalliedde described a group of patients who developed respiratory failure between 1 and 4 days after poisoning when they were otherwise relatively stable and free of muscarinic signs and coined the term “intermediate syndrome” (IMS).^6^ This syndrome overlapped extensively with the “Type II paralysis” described in 1974 by Wadia.^7^,^12^ Wadia also referred to “Type I paralysis”, early weakness with pyramidal signs, miosis, and impaired consciousness. Both papers described the electrophysiological signs associated with IMS/Type II paralysis. We also found these in our late respiratory failure group: sequential electrophysiological abnormalities indicating progressive neuromuscular transmission failure. Patients initially developed decrement–increment patterns at high-frequency stimulations. With clinical deterioration, decrement–increment patterns were progressively observed at intermediate and low frequencies, and at high frequencies a combination of decrement–increment and repetitive fade pattern was observed. Progressive decrement patterns were observed in patients who were ventilated for respiratory paralysis. The degree of muscle weakness correlated well with the electrophysiological features during clinical progression of IMS.^11^

In this study, for the first time, we describe the electrophysiological correlates of early respiratory failure in OP poisoning. This showed that the early respiratory failure is not associated with the same neuromuscular transmission defects seen in IMS.

None of these abnormalities was observed in those who developed early respiratory failure. This strongly suggests that this early respiratory failure does not involve neuromuscular transmission failure. The patients who developed early respiratory failure had relatively severe muscarinic features and also usually a low GCS suggesting CNS involvement and fitted the description of patients Wadia et al.^12^ referred to as having “Type I paralysis”. They stated this was a mus-carinic/CNS toxic syndrome and it may well be that they observed the lack of electrophysiological findings in early respiratory failure but only documented the positive findings in their reports.

This study provides support for the conclusions from the animal studies of Bird et al.^13^ and the speculations of earlier studies of respiratory failure in humans^7^,^5^ that early respiratory failure is primarily due to CNS and muscarinic effects. The study reiterates the importance of early and rapid titration of anti-muscarinic agents such as atropine.^14^ There is also a need for investigation of other antidotes, which may be more effective in the CNS as all these patients developed respiratory failure in spite of receiving atropine. There is also a need for antidotes, which protect the pre- and post-synaptic molecular targets of OP in the neuromuscular junction that underlie late respiratory failure. While RNS features may be an appropriate surrogate outcome for such studies, a validated means to measure muscarinic and CNS related respiratory dysfunction is needed to facilitate the development of better antidotes for the early syndrome.

Our study had some limitations. The number of patients who developed respiratory failure was small, presumably because of improved care. Chlorpyrifos was the OP agent in the majority and other different types of OPs are not adequately represented in this study. Since it is believed that different OPs differ in their clinical profile and response to oximes^15^ the frequency, time-course and manifestations of early and late respiratory failure may vary accordingly. Although to some extent this was an advantage in seeing two clearly distinct patterns.

RNS studies were performed only on distal muscles although the diaphragm may be more indicative of the underlying pathophysiology. However, these RNS findings did correlate reasonably well with features of IMS in our series. Phrenic nerve conduction studies are not frequently done because the technique is less precise and standardized than peripheral nerve conduction studies, and they are more difficult to conduct. The technique has recently been revised by Bolton and colleagues^16^ with promising results. They measured the reproducibility of the diaphragm compound action potential after electrical stimulation of the phrenic nerve and found it had the same accuracy as the thenar compound action potential after electrical stimulation of the median nerve.^16^ Phrenic nerve conduction studies have been conducted in OP poisoned patients with late respiratory failure by Singh et al.^17^ They did not do concurrent RNS studies, so it is not clear whether the phrenic nerve conduction studies were better than peripheral RNS studies in following the pathophysiology. Ideally, to study the diaphragmatic neuromuscular junction, serial phrenic nerve RNS studies would need to be performed in acutely poisoned patients. This has not been done before and may not be practical.

## Conclusions

The normal RNS in all seven patients who developed early respiratory failure suggests that the mechanism is muscarinic and/or central and not due to neuromuscular junction failure. This re-emphasizes the need for early rapid atropinisation. Further studies are needed to quantify CNS and muscarinic dysfunction to assist in the development of better treatments for the severe and early OP poisoning.
